# Supporting our sustainable future: a shared decision-guiding principle

**DOI:** 10.1093/nsr/nwag572

**Published:** 2026-09-04

**Authors:** Junze Zhang, Bojie Fu, Nan Zhang, Prajal Pradhan, Antoinette Tordesillas, Michael E Meadows, Keith R Skene, Xutong Wu, Robin Batterham, Daniel Bilbao, Alexis T Bell, Marc-Olivier Coppens, Peter Cummings, Peter P Edwards, Keith E Gubbins, Jinghai Li, Raffaella Ocone, Daya Reddy, Philipp Rudolf von Rohr, Bernd Sachweh, Richard Williams, Ning Yang, Gavin Costigan, Carlos Henrique de Brito Cruz, Peggy Oti-Boateng

**Affiliations:** State Key Laboratory of Regional and Urban Ecology, Research Center for Eco-Environmental Sciences, Chinese Academy of Sciences, China; State Key Laboratory of Regional and Urban Ecology, Research Center for Eco-Environmental Sciences, Chinese Academy of Sciences, China; State Key Laboratory of Mesoscience and Process Engineering, Institute of Process Engineering, Chinese Academy of Sciences, China; Integrated Research on Energy, Environment and Society, Energy and Sustainability Research Institute Groningen, University of Groningen, Netherlands; Potsdam Institute for Climate Impact Research, Germany; School of Mathematics and Statistics, University of Melbourne, Australia; School of Geography and Ocean Sciences, Nanjing University, China; Department of Environmental & Geographical Science, University of Cape Town, South Africa; The Living Laboratory, University of Dundee, UK; State Key Laboratory of Earth Surface Processes and Hazards Risk Governance, Faculty of Geographical Science, Beijing Normal University, China; Department of Chemical Engineering, University of Melbourne, Australia; ExxonMobil Technology and Engineering Company, USA; Department of Chemical and Biomolecular Engineering, University of California, USA; Department of Chemical Engineering and Centre for Nature-Inspired Engineering, University College London, Berkeley, UK; School of Engineering & Physical Sciences, Heriot-Watt University, UK; Department of Chemistry, University of Oxford, UK; Department of Chemical and Biomolecular Engineering, North Carolina State University, USA; State Key Laboratory of Mesoscience and Process Engineering, Institute of Process Engineering, Chinese Academy of Sciences, China; School of Energy, Geoscience, Infrastructure and Society, Heriot-Watt University, UK; Department of Mathematics and Applied Mathematics, University of Cape Town, South Africa; Department of Mechanical and Process Engineering, ETH Zurich, Switzerland; Department of Mechanical and Process Engineering, RPTU University Kaiserslautern-Landau, Germany; Heriot-Watt University, UK; State Key Laboratory of Mesoscience and Process Engineering, Institute of Process Engineering, Chinese Academy of Sciences, China; The Foundation for Science and Technology, UK; The Research Networks at Elsevier, UK; The African Academy of Sciences, Kenya

Adopted in 2015 to succeed the Millennium Development Goals (MDGs), the 17 Sustainable Development Goals (SDGs) universalized the transformation agenda for all countries, whether rich or poor, pledging to ‘Leave no one behind’ [1]. As the 2030 deadline approaches, that pledge is faltering. Poverty and hunger are rising again, greenhouse-gas emissions remain near record highs and confidence in multilateral action is weakening [2]. Accelerating progress toward the SDGs is therefore a global priority.

Achieving the SDGs means steering a complex science–technology–engineering–policy system that must build social foundations and prosperity within planetary boundaries across local-to-global scales [3]. Sustainability-transition, transformation and just-transition research has demonstrated that such change is systemic, contested and deeply shaped by place-specific conditions rather than a process of optimizing isolated economic, social or environmental objectives [4,5]. However, recognizing complexity does not by itself provide guidance for action. Decision-makers still need to navigate competing priorities, allocate limited resources and determine how different objectives should be reconciled across contexts [6]. What remains lacking is a shared decision-guiding principle that can translate scientific understanding of sustainability transformations into practical choices while respecting diverse pathways.

The next SDG Summit in 2027 will be the last before 2030 and is expected to open formal discussions on the post-2030 agenda [7]. Sustainable development, however, cannot be tied to a fixed endpoint, making it important that such discussions are grounded in principles that remain valid beyond 2030. We therefore propose compromise-in-competition (CIC) as a shared decision-guiding principle, rather than a universal substantive blueprint, for accelerating the SDGs and informing the post-2030 agenda.

## THE PRINCIPLE OF COMPROMISE-IN-COMPETITION

The principle of CIC originates from decades of research on complex systems in engineering and science. It was first recognized in gas–solid fluidization, where two physically dominant mechanisms coexist and compete. Particles tend to minimize their potential energy, whereas the fluid tends to minimize its flow resistance, and neither tendency can fully prevail [8]. Their compromise generates structures at the mesoscale, the intermediate scale between microscopic particles and the macroscopic system, and these mesoscale structures dominate the system’s behavior [9]. Formulating this CIC as a multi-objective optimization problem, as realized in the energy-minimization multi-scale model, greatly improved computational accuracy and scalability [8]. The same logic was subsequently identified across many disparate fields, leading to the broader concept of mesoscience, which attributes the complexity of systems at different levels to compromise in competition between their dominant mechanisms.

In CIC, competition refers to the coexistence of at least two dominant mechanisms or tendencies, each approaching its own extremum. Compromise, understood as coordination, refers to their mutual constraint, such that no single tendency can be maximized or minimized independently and the system settles into a state that dynamically balances the competing tendencies [8]. As the relative importance of competing mechanisms shifts with changing conditions, the resulting compromise is inherently dynamic rather than static. It enables adaptation, but it also generates structural complexity and regime transitions. Complexity is thus not noise to be eliminated but the predictable outcome of competing dominant tendencies that must be coordinated rather than optimized in isolation.

## WHY SUSTAINABILITY GOVERNANCE NEEDS CIC

Sustainability governance faces an analogous challenge in translating the SDGs from a shared global vision into effective action. The SDGs provide a common normative framework for sustainable development from 2016 to 2030, but their value ultimately depends on how these aspirations are interpreted, prioritized and implemented in practice. National and subnational governments are the principal actors responsible for translating global commitments into policies, investments, institutions and public services, while operating under different resource constraints, development conditions, institutional capacities and societal priorities [5].

Existing research on SDG governance has recognized that progress depends on reconciling competing objectives rather than pursuing any single objective in isolation, and several research traditions have established that sustainable development is systemic, contested and place based [3,10]. Planetary-boundary research delineates the biophysical limits within which humanity can safely operate, and safe-and-just-space frameworks couple these ecological ceilings with the social foundations required to meet basic human needs [11,12]. Nexus approaches analyze the interdependence of resource systems such as water, energy and food, warning that interventions in one sector can shift pressures onto others [13]. Transition and transformation research examines how technologies, institutions and behaviors co-evolve through contested, path-dependent processes and just-transition research asks who bears the costs of such change and how burdens and benefits are distributed [4]. Policy-coherence research, finally, investigates how objectives interact across sectors and levels of governance and how contradictions between them can be reduced [5].

Research on SDG interactions has further demonstrated that these interdependencies are dynamic rather than fixed [14,15]. Studies increasingly show that relationships among SDGs can involve facilitation, inhibition and neutral effects, with their direction and strength varying according to the intervention, context and governance level [16,17]. In particular, causal relationships between SDGs can change across spatial scales, such that an interaction that appears facilitating at the global or regional scale may become neutral or inhibiting in specific national contexts [16]. These findings highlight the importance of accounting for cross-scale dependencies, feedbacks, time lags and potentially nonlinear responses when evaluating pathways toward sustainable development.

**Figure 1.**
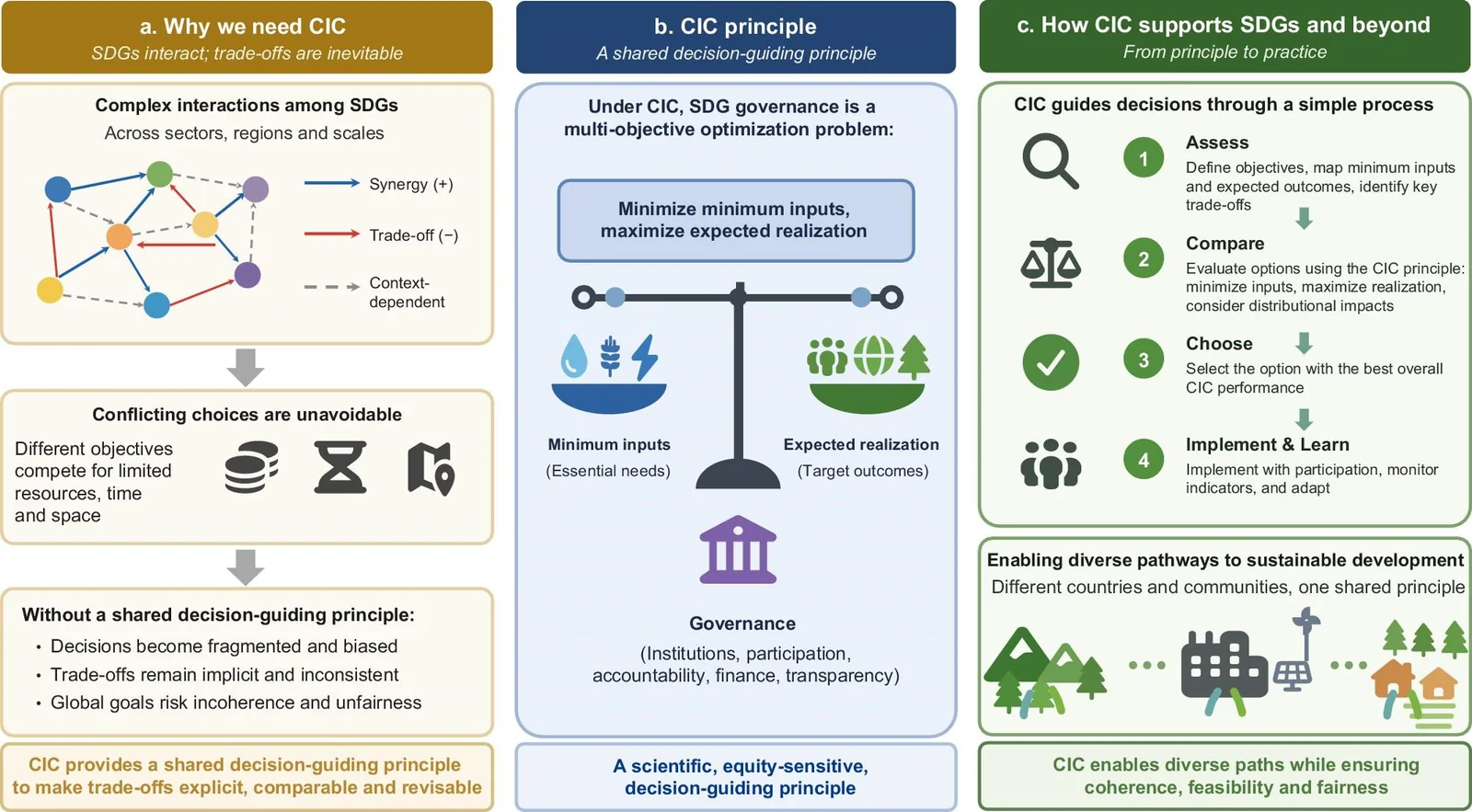
The CIC principle for navigating the governance of SDGs and enabling diverse pathways beyond 2030. (a) Rationale: SDGs are highly interdependent across sectors, regions and scales, producing inevitable trade-offs; without a shared principle, decisions become fragmented, inconsistent and potentially unfair. (b) Principle: CIC is a shared decision-guiding principle that seeks to minimize the minimum inputs required to meet Essential Needs and maximize the expected realization of Target Outcomes, within a governance context. (c) Application: CIC guides a transparent decision-making process—assess, compare, choose and implement & learn—that supports diverse pathways while maintaining coherence, feasibility and equity.

What these frameworks seldom specify is how competing objectives should be reconciled when a concrete decision must be made. SDG implementation is shaped by sectoral interdependencies, resource constraints and differences across places and governance levels, so an intervention that generates benefits in one context or at one scale may create pressures or trade-offs elsewhere. Without a shared decision-guiding principle, such challenges can lead to fragmented choices, hidden trade-offs and inconsistent priorities across countries and governance levels, weakening policy coherence and potentially shifting environmental and social risks across places or into the future (Fig. 1a).

CIC addresses this implementation gap by supplying a shared decision-guiding principle in which rival claims on limited resources, values and attention (competition) are reconciled through transparent, revisable coordination (compromise) [8]. This approach does not seek to eliminate trade-offs or impose identical choices, but to make them explicit, comparable and accountable, so that progress toward some objectives is not achieved by silently sacrificing others. CIC is therefore not a substitute for existing frameworks but a cross-level, decision-guiding principle for navigating SDG governance while leaving context-specific pathways open. Its application to sustainability governance is developed in the next section.

## HOW CIC CAN SUPPORT THE SDGS AND BEYOND

Under CIC, SDG governance can be understood as a science-guided multi-objective optimization process that seeks to minimize resource inputs while maximizing desired sustainability outcomes under ecological, social and economic constraints (Fig. 1b). The SDGs compete implicitly for finite resources, fiscal space, political attention and ecosystem capacity, and accelerating one goal can constrain others [14]. Because no goal can be maximized in isolation, decision-making should expose trade-offs, compare candidate portfolios by their distributional outcomes per unit of input and record how settlements are reached and by whom [10]. Compromise should therefore be understood as an ongoing governance process rather than a one-off settlement. Technologies, societal values, environmental conditions and resource constraints continually alter the relative importance and consequences of competing claims. The resulting compromises must consequently be periodically re-evaluated, allowing classifications, priorities and thresholds to be revised as new evidence emerges and societal preferences evolve. In this way, CIC turns trade-off management into an object of systematic scientific analysis and transparent governance, and it can be formalized as a shared decision-guiding principle (Fig. 1c).

Applying this principle to SDG governance analysis requires distinguishing which SDGs measure inputs, which measure outcomes and which measure the governance that mediates between them. The conventional three-pillar view of economic, social and environmental dimensions has shaped SDG classification, but it cannot capture the multidimensional character of individual goals and their interdependencies [18]. CIC therefore suggests grouping SDGs into three functional categories, comprising Essential Needs, Target Outcomes and Governance [19]. Essential Needs are the material, energy, land, water, infrastructure and ecological inputs required to satisfy basic needs. The pertinent question is not whether their absolute consumption is minimal, but whether their rate of consumption is falling relative to the rate at which Target Outcomes improve. Target Outcomes are achieved improvements in human well-being, equity, resilience, ecological integrity and adaptive or regenerative capacity. Governance comprises the institutions, rules, data, finance, participation and accountability mechanisms that allocate costs and benefits and enable collective learning. These categories are functional rather than value neutral. What counts as a need, an outcome or governance arrangement must be justified in context.

With these categories in place, CIC can be stated as a scale-crossing decision-guiding heuristic (Fig. 1c). Among feasible options, the preferred portfolio is the one that delivers the greatest verified and equitably distributed improvement in Target Outcomes per unit of Essential-Needs input, prioritizing groups most deprived or exposed, while Governance documents who gains, who loses and how conflicts are resolved. Identifying such portfolios is not a task for decision-makers alone. Scientists from multiple disciplines should lead the analysis, bringing together complementary expertise to quantify input–outcome relationships, distributional incidence and trade-offs among goals, while decision-makers, affected communities and other stakeholders participate in setting priorities, assigning weights and agreeing on acceptable thresholds [20]. CIC is thus a decision-guiding framework and heuristic with normative commitments to equity and planetary boundaries, not a scientific law that predetermines what each place should value [8].

Governance in this sense must give affected groups standing, record unresolved disagreements, compensate or redesign options when benefits bypass vulnerable populations, and refer conflicts that cannot be settled at one level to higher-level interfaces. The same principle can therefore be applied across different regions and governance scales, from local to global, without requiring identical choices, while supporting coordination across sectors, regions and scales and leaving plural ends and implementation routes open.

Previous research has classified the SDGs at the goal level into Essential Needs, Target Outcomes and Governance [19]. However, this goal-level classification does not necessarily translate consistently to the SDG targets, particularly to their indicators, because individual indicators may capture different functional attributes. An SDG that is broadly characterized as an Essential Need, for example, may contain indicators that measure achieved improvements in well-being, equity or ecological integrity and therefore meet the criteria for Target Outcomes. This distinction is important because the same goal can encompass both resource inputs and achieved outcomes, as illustrated by SDG14 and SDG15, which include indicators related to resource use as well as biodiversity protection [19]. CIC therefore applies the three-category logic at the indicator levels, where classification is based on the dominant function of each measure rather than inherited mechanically from its overarching SDG.

The classification of SDG indicators should be operational, participatory and revisable. National or regional statistical offices, sectoral experts and affected communities should assign each measure according to its dominant function, using the questions in Table 1 as screening criteria. Indicators that capture both inputs and outcomes may carry multiple classifications, which should be documented rather than suppressed. Disagreements should be made explicit together with the criteria applied, reviewed through science–policy interfaces (SPIs), and revisited periodically as evidence, boundary conditions and societal values evolve. CIC does not therefore prescribe a single fixed classification for all contexts. Instead, it provides a heuristic through which each country or region can develop a differentiated classification, allowing the same indicator to function as an Essential Need in one context and a Target Outcome in another. Table 1 consequently illustrates the classification logic rather than assigning SDG indicators once and for all.

**Table 1.** Indicator-level classification of SDGs based on the CIC principle.

Category	Classification question	Illustrative SDG indicators	Multi-classing note
Essential Needs	Does the indicator measure the rate at which material, energy, land, water, infrastructure or ecological inputs are consumed relative to the improvement of Target Outcomes?	6.4.1 water-use efficiency; 7.2.1 renewable-energy share; 12.2.2 domestic material consumption	Also assess distributional access and ecological ceilings
Target Outcomes	Does it measure achieved well-being, equity, resilience, ecological integrity or adaptive and regenerative capacity?	1.2.2 multidimensional poverty; 2.1.2 food insecurity; 3.8.2 catastrophic health expenditure; 15.1.2 protected important biodiversity sites	Link outcome gains to input use and affected groups
Governance	Does it measure rules, capacities, participation, accountability, finance or data?	16.7.2 inclusive decision-making; 17.13.1 macroeconomic dashboard; 17.18.3 funded national statistical plans	Capture who decides, who benefits and how conflicts are resolved

Note: Classifications of SDG indicators are contextual and may overlap. This table illustrates CIC-inspired classification logic rather than prescribing a fixed assignment, and multi-classification of SDG indicators should be documented rather than forced into a single category.

The same analytical procedure applies beyond any single type of region, wherever development is locked into a persistent challenge. In resource-rich regions, the resource curse can arise when extraction generates revenue but crowds out diversification and leaves communities with depleted land and water. CIC treats the inputs that such extraction consumes, including land, water, energy and infrastructure, as Essential Needs, treats durable outcomes such as quality jobs, public health and ecological integrity as Target Outcomes, and requires Governance to document how revenues are shared and reinvested, so that

portfolios are compared by the outcomes they deliver per unit of input rather than by output alone. In resource-poor regions facing a poverty trap, the same principle protects the inputs needed to meet basic needs while directing investment toward the largest verified improvements in well-being, with Governance recording who benefits and who bears the costs. In both cases, CIC compares portfolios by distributional outcome per unit input and requires redesign when apparent gains bypass vulnerable groups.

## RECOMMENDATIONS AND CONCLUSION

Ahead of the 2027 SDG Summit, three instruments could test and refine CIC without treating it as a fixed universal template.

First, region-based CIC Implementation Roadmaps should translate the CIC principle into context-specific development strategies by integrating systems modeling, scenario analysis, stakeholder deliberation and data-driven assessment. Unlike conventional SDG implementation plans or voluntary national reviews that primarily track progress toward predefined targets, CIC principle would evaluate development pathways according to how efficiently resource inputs are converted into equitable and sustainable outcomes [8]. Each roadmap should therefore establish a minimum open-data infrastructure that integrates three interconnected dimensions, including Essential Needs related to resource use, energy, land and material inputs; Target Outcomes concerning well-being, equity, resilience and ecological integrity; and Governance mechanisms encompassing participation, accountability, finance and data transparency.

Additional requirements should include distributional impact assessments, ecological ceilings and a public trade-off register documenting who benefits, who bears costs and how unresolved conflicts are addressed. National statistical offices, planning and finance ministries, sectoral agencies and local governments should share custodianship, while independent audits should verify data quality, methodological assumptions and compliance with CIC procedures.

Second, standing SPIs at local, national, regional and global scales should translate evidence into actionable decisions. Beyond conventional knowledge-transfer functions, CIC-based SPIs should co-produce indicator classifications, evaluate whether policy choices reduce resource inputs while improving outcomes and assess whether trade-offs are transparently negotiated. They should include interdisciplinary researchers, statistical offices, policymakers, affected communities, and private-sector and civil-society actors, supported by clear mandates, conflict-of-interest rules and public documentation. Regular reviews should ensure that data access, model assumptions and value judgments remain transparent and open to revision.

Third, adaptive-management frameworks should maintain CIC as a learning process rather than a static assessment tool. They should combine real-time monitoring, feedback loops and scheduled revisions to update indicators, thresholds and model assumptions as conditions change. AI-enabled tools could integrate citizen science, Earth observation and administrative data to identify emerging trade-offs and evaluate alternative pathways, but their application should remain auditable, privacy-preserving, bias-tested and subject to public oversight. The decision principle itself should remain stable, whereas its operational parameters should evolve through trigger-based reviews and continuous learning.

Together, these instruments position CIC as a practical, equity-sensitive heuristic for guiding sustainable development toward 2030, 2100 and beyond. Its value lies not in eliminating diversity or political conflict, but in making trade-offs explicit, comparable and revisable across scales. Scientific guidance can discipline these choices, but realizing sustainable development will ultimately require governments, businesses and civil societies to negotiate responsibilities and act inclusively.

However, CIC should be understood within its limits. As a decision-guiding principle, it cannot by itself guarantee alignment across governance levels, resolve distributional conflicts, redress historical responsibilities, close financing gaps or correct power asymmetries, nor can it transform sustainable development into a purely solvable optimization problem. Its effective application therefore depends on broad interdisciplinary collaboration, bringing together expertise from the natural, social, economic and policy sciences to assess complex interactions, quantify trade-offs and evaluate distributional consequences. Its contribution is procedural, enabling trade-offs to become explicit, comparable and revisable across regions and scales while clarifying who benefits, who bears costs and how conflicts are addressed, thereby bridging interdisciplinary scientific evidence and political negotiation.

## References

[1] United Nations . Transforming Our World: The 2030 Agenda for Sustainable Development. New York: United Nations, 2015.

[2] Sachs JD, Lafortune G, Fuller G et al. Implementing Sustainable Development: 2030 and Beyond. Sustainable Development Report 2026. Paris/Dublin: SDSN/Dublin University Press, 2026.

[3] Luo L, Zhang J, Wang H et al. Innov Geosci 2024; 2: 100087. 10.59717/j.xinn-geo.2024.100087

[4] Davelaar D . Sustain Sci 2021; 16: 727–47. 10.1007/s11625-020-00872-0

[5] Independent group of scientists appointed by the United Nations Secretary-General . Global Sustainable Development Report 2023: Times of Crisis, Times of Change: Science for Accelerating Transformations to Sustainable Development. New York: United Nations, 2023.

[6] Di Lucia L, Slade R, Khan J. Nat Sustain 2022; 5: 131–8. 10.1038/s41893-021-00819-y

[7] United Nations . Pact for the Future, Global Digital Compact and Declaration on Future Generations. New York: United Nations, 2024.

[8] Li J . Proc R Soc A: Math Phys 2024; 480: 20240031. 10.1098/rspa.2024.0031

[9] Huang W, Li J, Edwards PP. Natl Sci Rev 2018; 5: 321–6. 10.1093/nsr/nwx083

[10] Barbier EB, Burgess JC. Economics 2017; 11: 1–22. 10.5018/economics-ejournal.ja.2017-28

[11] Rockström J, Donges JF, Fetzer I et al. Nat Rev Earth Environ 2024; 5: 773–88. 10.1038/s43017-024-00597-z

[12] Fanning AL, Raworth K. Nature 2025; 646: 47–56. 10.1038/s41586-025-09385-1 PMC12488500 41034533

[13] Liu JG, Hull V, Godfray HCJ et al. Nat Sustain 2018; 1: 466–76. 10.1038/s41893-018-0135-8

[14] Khot UA, Warchold A, Pradhan P. Environ Impact Assess Rev 2026; 118: 108274. 10.1016/j.eiar.2025.108274

[15] Zhang J, Sun W, Pradhan P et al. Environ Impact Assess Rev 2025; 115: 107990. 10.1016/j.eiar.2025.107990

[16] Wu K, Zhang J, Pradhan P et al. Sustain Prod Consum 2026; 67: 384–97. 10.1016/j.spc.2026.07.007

[17] Wu X, Fu B, Wang S et al. Nat Sustain 2022; 5: 452–9. 10.1038/s41893-022-00868-x

[18] Hametner M . Ecol Econ 2022; 199: 107490. 10.1016/j.ecolecon.2022.107490

[19] Fu B, Wang S, Zhang J et al. Natl Sci Rev 2019; 6: 386–8. 10.1093/nsr/nwz038 PMC8291389 34691883

[20] Malekpour S, Allen C, Sagar A et al. Nature 2023; 621: 250–4. 10.1038/d41586-023-02808-x 37704766

